# A modified MRI protocol for the increased detection of sacrococcygeal fractures in patients with thoracolumbar junction fractures

**DOI:** 10.1038/s41598-021-85167-9

**Published:** 2021-03-11

**Authors:** Eun Kyung Khil, Il Choi, Jung-Ah Choi, Young Woo Kim

**Affiliations:** 1 Department of Radiology, Hallym University Dongtan Sacred Heart Hospital, Hwaseong-si, Gyeonggi-do South Korea; 2 Department of Neurological Surgery, Hallym University Dongtan Sacred Heart Hospital, Hwaseong-si, Gyeonggi-do South Korea; 3 Department of Orthopeadic Surgery, Hallym University Dongtan Sacred Heart Hospital, Hwaseong-si, Gyeonggi-do South Korea

**Keywords:** Musculoskeletal system, Diagnosis, Medical imaging, Quality of life, Pain, Health care, Medical research, Risk factors, Signs and symptoms

## Abstract

This study aimed to identify concurrent thoracolumbar junction (TLJ) and sacrococcygeal (SC) fractures using a modified MRI protocol and analyze the risk factors associated with tandem fractures. We retrospectively investigated patients with MRI-confirmed TLJ fractures from January 2017 to March 2020. Patients were divided into two study groups: study 1 with a modified MRI protocol and study 2 with a routine protocol. The modified protocol included an extended field of view of sagittal scans in L-spine MRI covering the full SC spine. In study 1, frequency of concurrent TLJ and SC fractures was investigated. And we analyzed risk factors and compared CT and MRI for detecting SC fractures. In study 2, co-occurrence of both fractures was investigated. A total of 176 and 399 patients with TLJ fractures were enrolled in study 1 and 2, then SC fractures were identified in 53 (30.14%) and 36 patients (9.02%), respectively. An axial loading trauma mechanism was a significant risk factor (Odds ratio 7.0, *p* < 0.001), and MRI was more sensitive than CT in detecting SC fractures (*p* < 0.002). Thus, a modified MRI protocol was useful to detect the high occurrence of SC fractures in TLJ fractures, which concurrent fractures increased by an axial loading mechanism.

## Introduction

The thoracolumbar junction (TLJ; T10-L2) is the most common site of thoracolumbar (TL) fractures, which account for approximately 50–60% of TL fractures^1,2^ and up to 90% of traumatic spine fractures^3,4^. TLJ is a transition zone of the TL spine with two different degrees of mobility and is clinically important^1,5^. Many TL spine fractures are caused by falls, and the most common type of fracture is a burst fracture^6,7^. Burst or compression fractures are known to result from axial loading at the vertebral body^7,8^, and sacral horizontal fractures have also been related to axial loading^9–11^. Therefore, if axial loading is the mode of injury of a TLJ fracture, a co-existing sacrococcygeal (SC) fracture can be assumed to be more likely. Some TLJ fractures are unstable, leading to neurologic deficits and long-term deformities^3,12,13^. However, SC fractures, including sacral insufficiency fractures (SIFs), are usually stable. Consequently, these are often underestimated or neglected in elderly patients by clinicians and even by radiologists^10,11,14–16^. However, clinically, SC fractures affect the quality of life of patients by inducing lower back pain and sacral pain. In rare cases, SC fractures cause neurologic deficits such as radiculopathy of lower extremities, bladder or bowel dysfunction^10,17,18^.

To date, the presence of concurrent SC and TL spine fractures has rarely been investigated, and its prevalence is reported to be 3.8–10.6%^15,19^. However, previous studies have not focused on acute injuries, trauma situations, or the TLJ itself, making it difficult to assess the association between TLJ and SC fractures.

Although CT is the gold standard for diagnosing TL fractures^20^, MRI is essential for the accurate diagnosis of spine fractures^20–23^. Routine MRI protocols are used for patients with trauma in common clinical settings. A routine L-spine MRI protocol, based on the American College of Radiology (ACR) guidelines, recommends including regions from T12 to S1 in a sagittal view^24^ and does not routinely include regions below S2. In particular, in the SC area, MRI is recommended after radiography rather than CT^25^. Our modified L-spine MRI protocol performed all sagittal scans with an extended field of view (FOV), including regions below S2, and used a scout view with fat saturation (FS) sequence to cover the regions above T11.

This study aimed to identify concurrent TLJ and SC fractures using the modified MRI protocol and analyze the risk factors associated with tandem fractures.

## Methods

### Study design and enrolment

This retrospective study included spine MRI data of patients who had been treated from January 2017 to March 2020 at our hospital. The data were extracted from the Picture Archiving and Communication System (PACS). Patients were divided into two study groups (study 1 and 2) based on the use of the modified MRI protocol. After a routine medical examination, patients with clinically suspected spinal fractures first underwent CT and then MRI to evaluate occult soft tissue injury. The study was approved by the Institutional review board by the Institutional Review Board of Hallym University Hospital, and the requirement of informed consent was waived due to the retrospective nature of the study. All the methods of this study were performed in accordance with the relevant guidelines and regulations.

### Study 1

**Figure 1:**
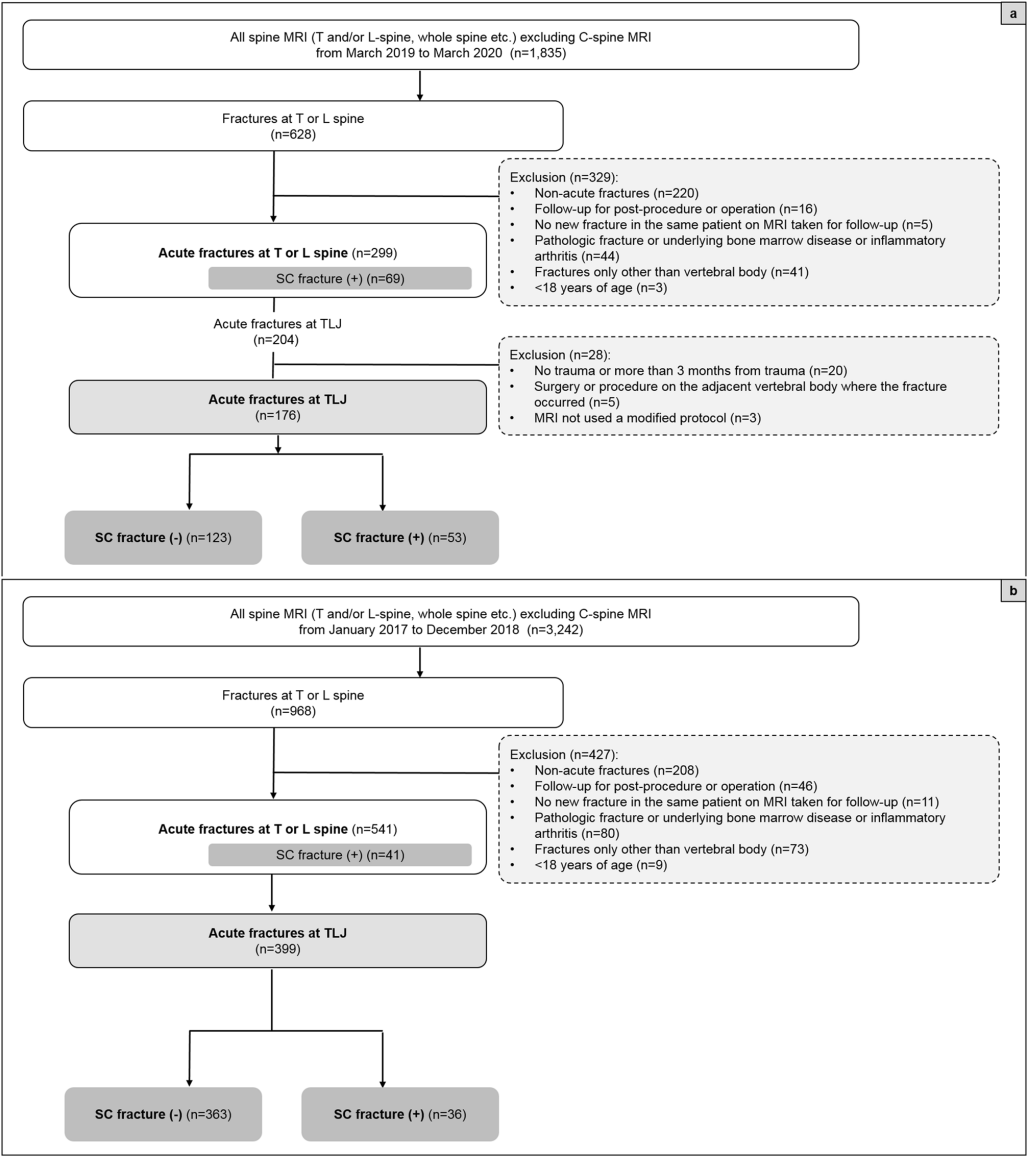
Study enrolment flow chart for patients in study 1 (**a**) and study 2 (**b**). *TLJ* thoracolumbar junction, *SC* sacrococcygeal.

The existing routine L-spine MRI protocol was modified and implemented from March 2019 to March 2020, to assess the frequency of concurrent TLJ and SC fractures on all spine MRI scans excluding those of the C-spine. The first set of exclusion criteria is described in Fig. 1a. Only adult patients with acute injuries were included: The hospital’s PACS was searched for spine MRI reports between March 2019 and March 2020, first using the search term “fracture” and subsequently, the terms “acute” and “recent”. Additionally, thoracolumbar injury classification and severity (TLICS) scores are reported for all the patients with acute TL spine fractures at the hospital; “TLICS” was therefore additionally included as a search term to find missing acute fractures. After applying the second set of exclusion criteria as described in Fig. 1a, enrolled patients were then classified into two groups according to the presence of SC fracture. Additionally, the SC fracture group was subdivided into two groups as follows: the upper SC fracture group (S1–S3; may be unstable) and the lower SC fracture group (S4 to coccyx; relatively stable)^10,17^.

### Study 2

In this group, the frequency of TLJ and SC fractures using the routine spine MRI protocol was assessed. The existing protocol acquired sagittal sequences covering T11–S2, sometimes using an extended FOV at the request of a clinician. For instance, the sagittal sequences were acquired using an extended FOV for a patient with an acute fracture at the SC spine who initially underwent CT. Fractures of the T or L-level spine were examined in all spine MRIs, excluding cervical spine MRIs, regardless of trauma, from January 2017 to December 2018. The exclusion criteria are shown in Fig. 1b.

### Routine and modified spine MRI protocols

**Figure 2:**
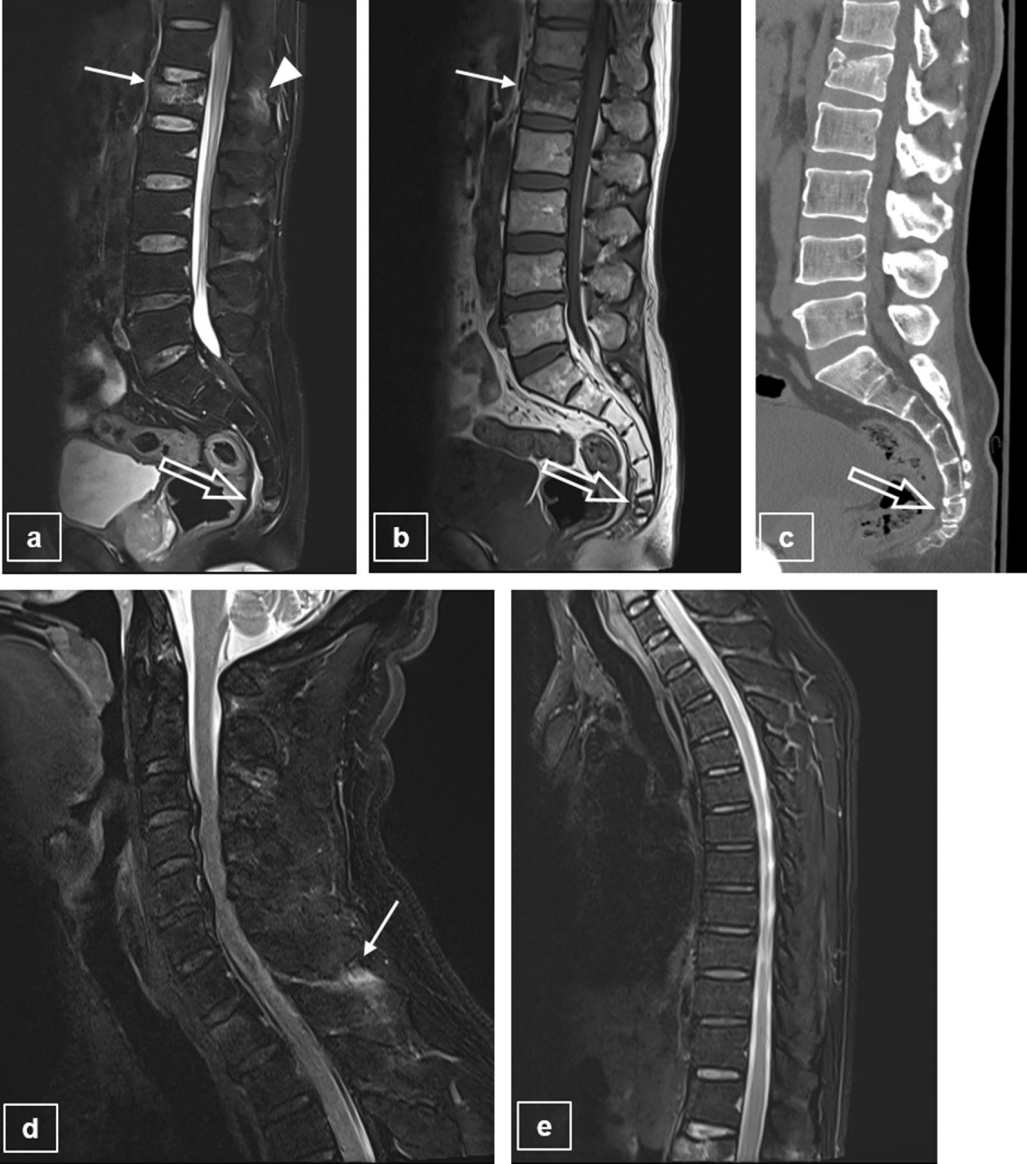
Example of concurrent fractures of the TLJ and SC regions on L-spine MRIs using the modified MRI protocol. Modified L-spine sagittal MRI scans with T2-WI (**a**) and T1-WI (**b**) show a recent compression fracture (arrow) at the L1 vertebral body with PLC injury (arrowhead), and a recent fracture at the coccyx upper body (open arrow), which is revealed on a modified L-spine CT scan (**c**). On scout scans with T2-WI fat saturated sequence of cervical and thoracic spines (**d**, **e**), there is no recent fracture, but high signal intensity change is seen in the interspinous ligament of C6/7, indicating ligament injury (arrow). *TLJ* thoracolumbar junction, *SC* sacrococcygeal, *WI* weighed image, *PLC* posterior ligamentous complex.

Two types of 3.0-T MRI scanners were used (Skyra or Verio, Siemens Healthcare, Erlangen, Germany) and the parameters of the L-spine MRI are shown in Table 1. The routine L-spine MRI protocol included sagittal and axial T1 and T2-weighted images, a scout view with T2-weighted images of cervical and thoracic spines, and an additional sagittal T2 FS sequence to assess the trauma. The modified MRI protocol was as follows: for adult trauma patients, the FOV of all sagittal scans in routine L-spine MRIs was changed (Fig. 2). The FOV was extended such that the upper limit would not change and the lower limit included the coccyx. To ensure an accurate assessment, whenever an SC fracture was suspected on sagittal scans, additional MRI scans were performed with following sequences: coronal T2 FS sequence parallel to the long axis of the SC spine, an axial T2-weighted sequence, and a plane perpendicular to the coronal plane. In addition, scout images of all spine MRIs were unified with T2 FS sequences; therefore, even if an L-spine MRI was acquired, a thoracic spine scan would be included. Similarly, if a thoracic spine MRI was acquired, the coccyx would be included in the L-spine scout view.

**Table 1:** Protocol of L-spine MRI, including modified sequences.

Type	L-spine MRI					C-spine scout	T-spine scout
Parameter	Sagittal	Sagittal	Sagittal	Axial	Axial	Sagittal	Sagittal
	T2-WI TSE	T1-WI TSE	T2-WI TSE FS	T2-WI TSE	T1-WI TSE	T2-WI TSE FS*	T2-WI TSE FS*
TR (ms)	2860–4410	416–567	3000–4920	3600–7710	418–788	3560–4380	3000–4340
TE (ms)	82 ~ 90	10	84	100	15	83 or 88	82 or 85
Matrix size	512 × 410	448 × 269	448 × 314	384 × 230	384 × 230	448 × 269	384 × 230
FOV (cm)	34 × 34 or 35 × 35*	34 × 34 or 35 × 35*	34 × 34 or 35 × 35*	16 × 16	16 × 16	22 × 22	32 × 32
Section thickness (mm)	3	3	3	4	4	3	3
Intersection gap (mm)	0.6	0.6	0.6	0.3	0.3	0.3	0.6
Echo train length	16 ~ 19	5	17 ~ 21	16	3	17	17
No. of signals acquired	2	1	2	2	2	2	2
Scan time (min)	1′ 50″–2′ 25″	1′30″–1′ 41″	2′ 51″–3′ 41″	2′ 17″–3′ 14″	2′ 27′–3′ 19″	2′ 30″–2′ 54″	2′ 15″

*TE *echo time, *TR *repetition time, *No *number, *FOV *field of view, *WI *weighted image, *TSE *turbo spin-echo, *FS *fat saturation.

Modified MRI protocol*: The previous 32 cm × 32 cm of FOV has been changed to 34 cm × 34 cm or 35 cm × 35 cm.

Moreover, the CT protocol was modified for patients with trauma in study 1, wherein spinal CT, including the L-spine, was performed, and the sagittal plane included the coccyx (Fig. 2).

### Spine MRI evaluation

All spine MRIs were reported by two musculoskeletal radiologists (with 5 and 12 years of experience respectively). Additionally, in study 1, a radiologist with 5 years of experience reviewed the MRIs to finalize the data to be included for the evaluation of risk factors of concurrent TLJ and SC fractures. For each TL fracture, a TLICS score was applied^7^. An SC fracture was defined as a fracture with a typical cortical disruption or fracture line, and a suspicious fracture line with combined bone marrow edema on T2 FS images.

### Assessment of risk factors for concurrent TLJ and SC fractures

When using the modified protocol, the following factors were assessed for risk associated with concurrent TLJ and SC fractures: age, sex, weight, height, body mass index (BMI), bone mass density (BMD), TLICS score, number of T or L fractures, and trauma mechanism. The TLICS score can be used as an indicator of the degree of injury. Trauma mechanism was classified into cases of axial load application to the sacral area (e.g., landing on the buttock due to a slip or fall) and cases of non-axial loading (e.g., various mechanisms such as a traffic accident, excessive use of the waist, back injury due to a sudden change in posture, etc.).

### Statistical analysis

In study 1, patients with TLJ fracture were classified into two groups based on the presence of SC fractures. For the demographic data, the chi-square test, and t-test, and Mann–Whitney U test were used to assess the categorical or continuous variables as appropriate. To analyze the statistically significant variables among the various risk factors, a binary logistic regression analysis was carried out. A multivariate logistic regression was additionally used for the correction of the multiple variables used. The modified protocol allowed for the evaluation of CT and MRI scans; the McNemar’s test was used to assess SC fractures in cases of TLJ fractures on CT and MRI scans after establishing MRI as the golden standard for assessing fractures. Additionally, the fracture rate was measured at each T and L spine level and the SC fracture rate was assessed using the test of marginal homogeneity. In study 2, the frequency of fractures at each level of the TLJ was charted using a frequency analysis.

SPSS Statistics 20.0 was used for most of the statistical analyses, and SAS software 9.4 for the test of marginal homogeneity in comparison between CT and MRI.

## Results

### Subjects and demographic characteristics

After applying the inclusion and exclusion criteria (Fig. 1), 176 adult patients with trauma (65 males and 111 females; mean age 64.31 ± 16.70 years) were enrolled in study 1 (scans performed with a modified MRI protocol) and 399 patients (176 males and 223 females; mean age 62.46 ± 21.30 years old) were enrolled in study 2 (scans performed with a routine protocol).

### Frequencies of concurrent TLJ and SC fractures

**Figure 3:**
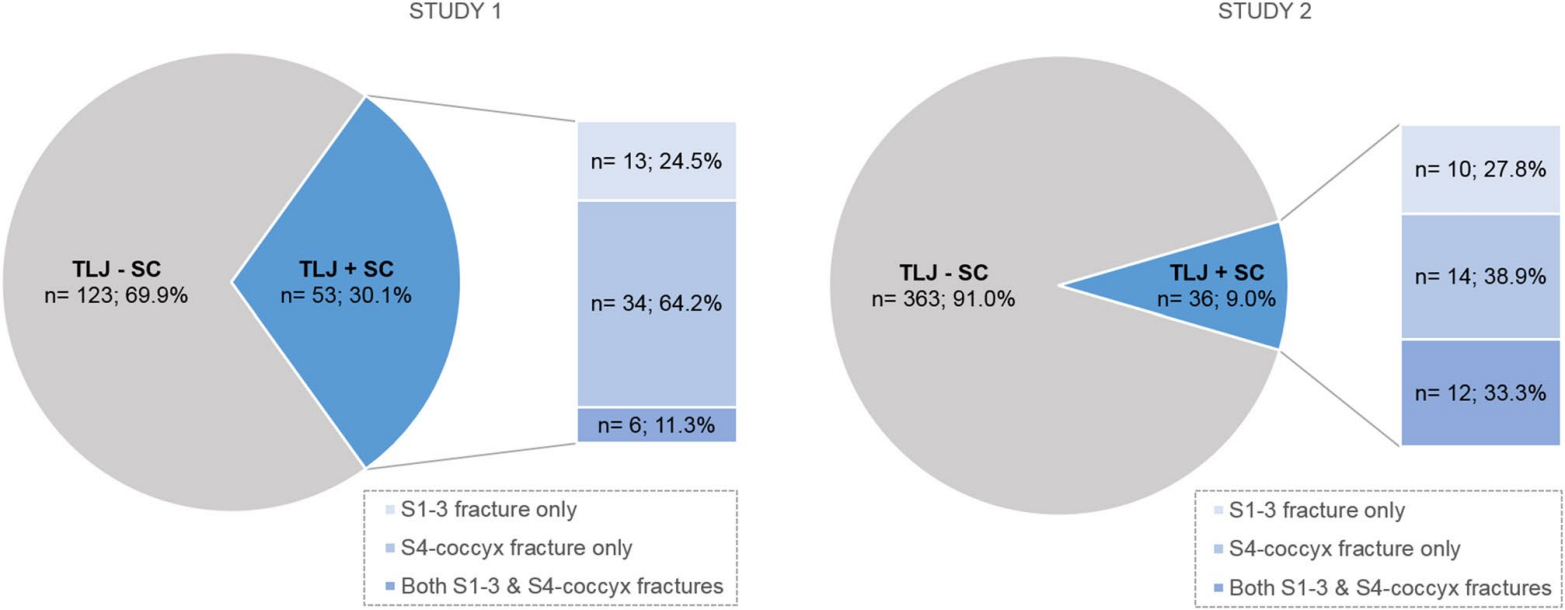
Frequencies of accompanied SC fractures in patients with TLJ fractures in study 1 and study 2, and fracture frequencies of the upper and lower portion in patients with SC fracture. *TLJ* thoracolumbar junction, *SC* sacrococcygeal.

Among the scans performed with the routine protocol from 2017 to 2018, 399 patients presented with acute TLJ fractures regardless of the trauma mechanism. Of these, SC fractures were found in 36 patients (9.02%) (Fig. 3). Among the 541 patients with acute T or L fractures, 41 patients had SC fractures (7.58%).

After applying the modified MRI protocol (from March 2019 to March 2020), 204 patients were found to have acute TLJ fractures (Fig. 1a). After applying the exclusion criteria, 28 patients were excluded, and 176 patients with acute traumatic TLJ fractures were available for evaluation. Among them, 53 patients (30.14%) had SC fractures (Fig. 3). The average interval between trauma and performed MRI was approximately 4 days (4.02) and that between CT and MRI was approximately one day (1.14). Among the patients with SC fractures, the SC fractures were higher in frequency in the S4-coccyx region than in the S1-3 region (Fig. 3). When demographic characteristics were compared between patients with TLJ fractures only (n = 123) and those with both TLJ and SC fractures (n = 53), a significant difference between the two groups was found only with regard to the trauma mechanism of axial loading (*p* < 0.001; Table 2). In addition, of the 101 TLJ fractures that occurred due to axial loading trauma, 45 had SC fractures (44.55%).

**Table 2:** Demographics and risk factors for sacrococcygeal fractures.

	TLJ only (n = 123)	TLJ + SC (n = 53)	*P*-value
**Sex**			
Male	46 (37.4%)	19 (35.85%)	0.845
Female	77 (62.6%)	34 (64.15%)	
**Age (years)**	63.88 ± 17.12	65.3 ± 15.82	0.605
< 50	25 (20.33%)	7 (13.21%)	0.261
≥ 50	98 (79.67%)	46 (86.79%)	
**BMI (kg/m**^**2**^**)**	23.76 ± 3.69	24.36 ± 3.58	0.328
< 18.5	5 (4.2%)	4 (7.55%)	0.282
18.5 ~ ≤ 25	73 (61.34%)	26 (49.06%)	
> 25	41 (34.45%)	23 (43.4%)	
Height (cm)	160.44 ± 9.43	160.26 ± 9.76	0.913
Weight (kg)	61.45 ± 12.69	62.92 ± 12.79	0.485
BMD (g/cm^2^)	0.905 ± 0.19	0.912 ± 0.20	0.824
**T score**	− 1.95 ± 1.56	− 1.93 ± 1.57	0.943
> − 2.5	69 (62.16%)	30 (57.69%)	0.586
≤ − 2.5	42 (37.84%)	22 (42.31%)	
**TLICS score**	2.89 ± 1.42	2.77 ± 1.37	0.583
1–3	78 (63.41%)	34 (64.15%)	0.926
≥ 4	45 (36.59)	19 (35.85)	
Number of fractures	1.12 ± 0.472	1.09 ± 0.295	0.694
**Trauma mechanism**			
Axial loading	56 (45.53%)	45 (84.91%)	< 0.001*
Non- axial loading	67 (54.47%)	8 (15.09%)	

*TLJ* thoracolumbar junction, *TLJ + SC* thoracolumbar junction and sacrococcygeal fractures, *BMI* body mass index, *BMD* bone mass density, *TLICS* Thoracolumbar Injury Classification and Severity; BMI was divided into low weight (< 18.5), normal (≥ 18.5 and ≤ 25), and overweight (> 25) groups as per the WHO classification. BMD was subdivided based on a cutoff threshold corresponding to a value under − 2.5 of the T-score, which is considered to be reflective of osteoporosis. TLICS values were divided into groups corresponding to 1–3 points for the group to receive a conservative treatment and ≥ 4 points for the group in which to consider surgery.

*p < 0.05*.*

### Risk factor analysis of TLJ and SC fractures when using the modified MRI protocol

An analysis of risk factors associated with concurrent TLJ and SC fractures was carried out (Table 3). The odds ratio (OR) was statistically significant only for a trauma mechanism of axial loading (OR 6.73, *p* < 0.001). The odds ratio for a trauma mechanism of axial loading remained significant (OR 7.0, *p* < 0.001) even after correcting for and analysing the variables in a multivariate analysis.

**Table 3:** Univariate and multivariate logistic regression analyses of risk factors for SC fractures in patients with TLJ fractures.

Factors	Univariate			Multivariable		
	OR	95% CI	*P*-value	OR	95% CI	*P*-value
**Sex**						
Male	0.935	0.479–1.828	0.845	1.339	0.569–3.153	0.504
Female	1			1		
**Age (years)**	1.005	0.986–1.025	0.603			
< 50	1			1		
≥ 50	1.676	0.676–4.158	0.265	1.282	0.452–3.639	0.641
**BMI (kg/m**^**2**^**)**						
< 18.5	2.246	0.56–9.008	0.253	1.447	0.329–6.363	0.625
18.5 ~ ≤ 25	1		0.287	1		0.342
> 25	1.575	0.799–3.106	0.190	1.721	0.824–3.595	0.148
Weight (kg)	0.998	0.965–1.033	0.912			
Height (cm)	1.009	0.984–1.035	0.483			
BMD (g/cm^2^)	1.216	0.221–6.671	0.822			
**BMD (T-score)**						
> − 2.5	1			1		
≤ − 2.5	1.205	0.616–2.356	0.586	1.183	0.521–2.685	0.688
**TLICS score**	0.943	0.746–1.192	0.623			
1–3	1			1		
≥ 4	0.969	0.495–1.894	0.926	1.024	0.471–2.226	0.952
**Trauma mechanism**						
Axial loading	6.730	2.93–15.458	< 0.001*	7.004	2.948–16.639	< 0.001*
Not axial loading	1			1		

*SC* sacrococcygeal, *TLJ* thoracolumbar junction, *OR* odds ratio, *CI* confidential index, *BMI* body mass index, *BMD* bone mass density, *TLICS* Thoracolumbar Injury Classification and Severity.

*p < 0.05.

### Differences between CT and MRI-based frequencies when using the modified MRI protocol

**Figure 4:**
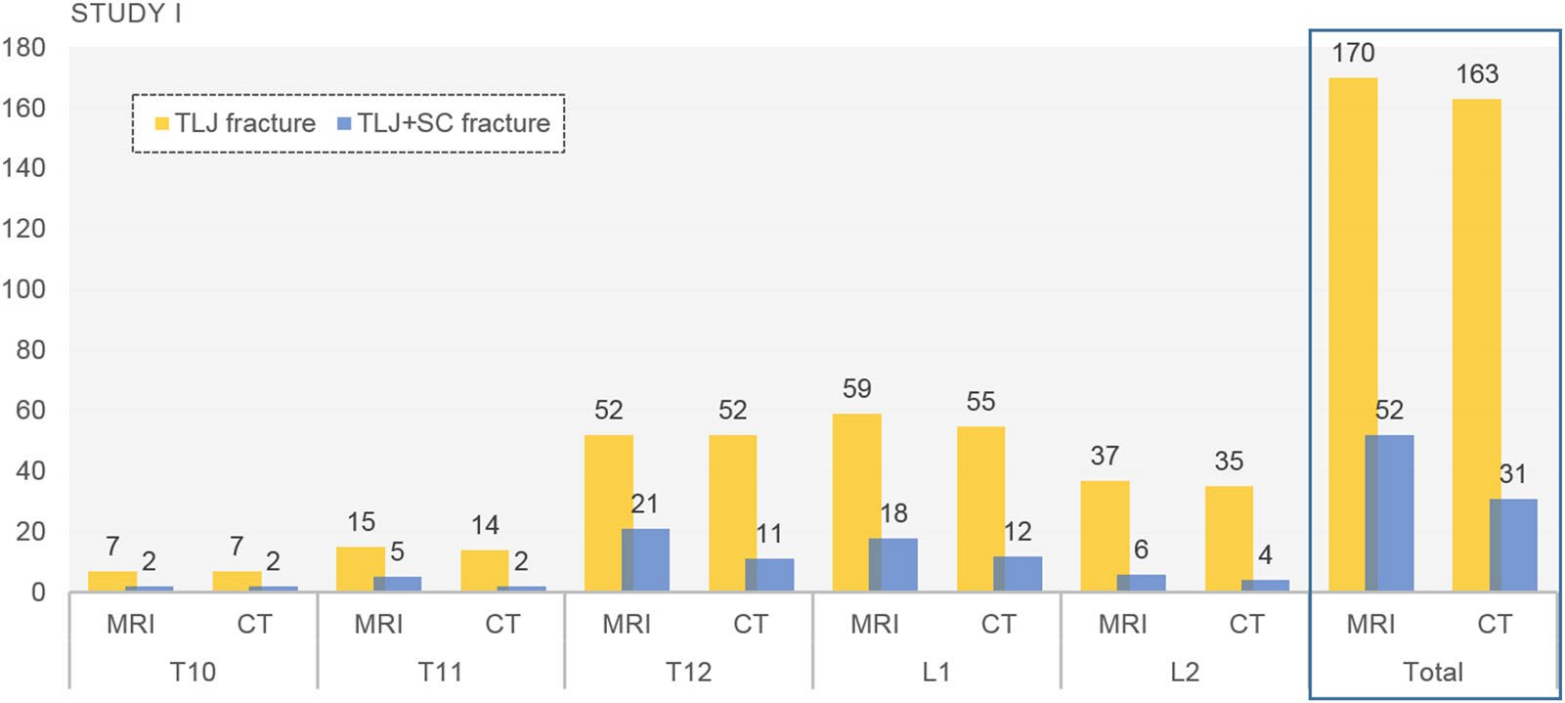
Comparison between CT and MRI in assessing the number of TLJ fractures and concurrent TLJ and SC fractures in study 1. *TLJ* thoracolumbar junction, *SC* sacrococcygeal, *TLJ + SC* thoracolumbar with sacrococcygeal.

**Figure 5:**
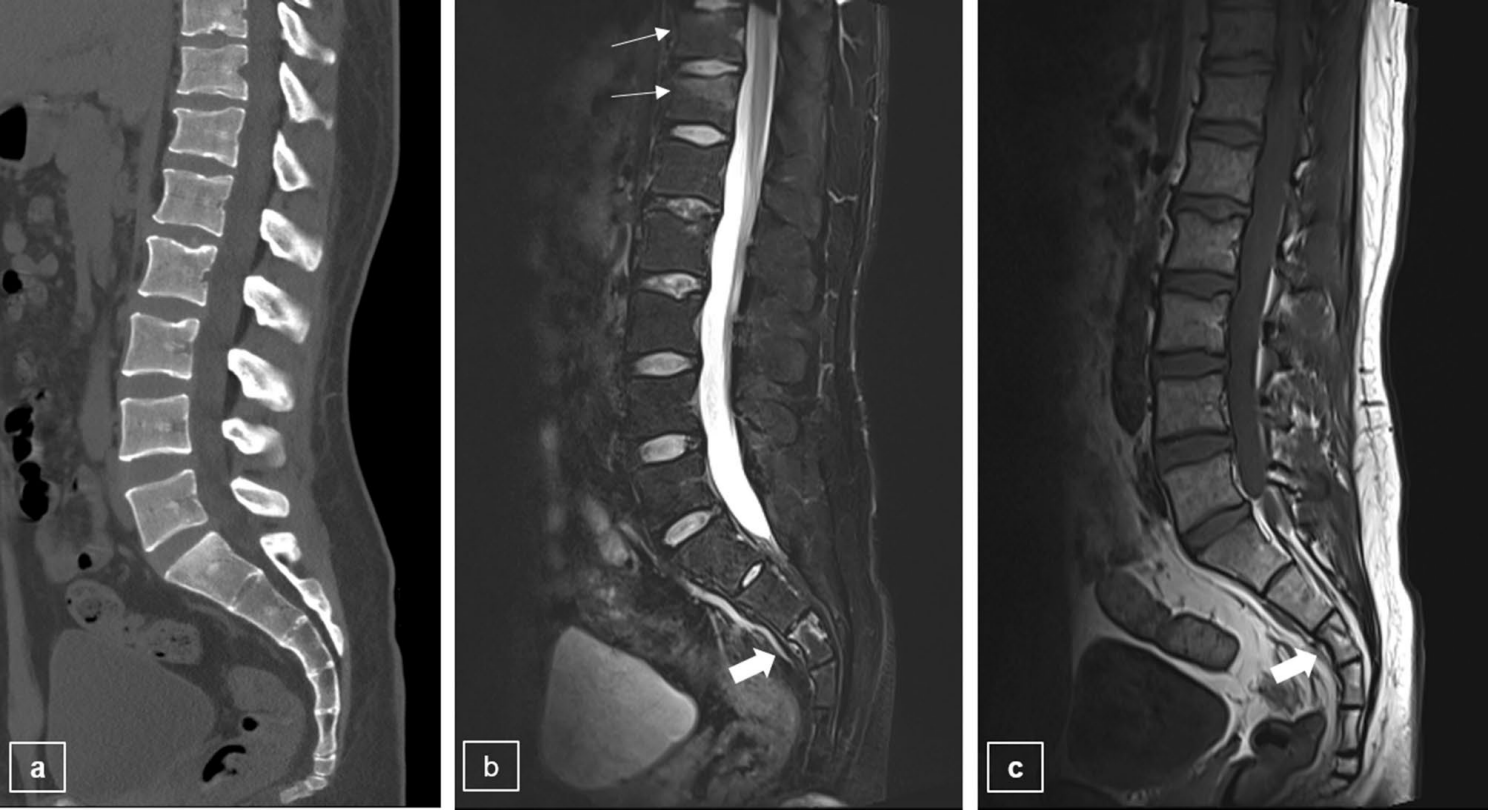
Patients with recent compression fractures at the vertebral bodies of T11 and T12. On modified L-spine CT (**a**), there is no definite acute fracture at the S3 body, or at T11 and T12. However, on the modified L-spine MRI with T2WI fat saturation (**b**) and T1WI (**c**), fractures are shown at the vertebral bodies of T11, T12 (thin arrows), and the upper portion of the S3 body (thick arrow).

Among 176 patients in study 1, there were 153 patients who underwent both CT and MRI, and 23 patients who did not undergo CT or whose CT did not include all SC levels were excluded. When using the modified MRI protocol, the method of detection to determine the frequency of TLJ fractures and SC fractures in CT and MRI scans was carried out as follows (Fig. 4, Table 4). The MRI scans reveals that there were 170 TLJ fractures in 153 patients with TLJ fractures, of which 47 patients had 52 SC fractures. Seven TLJ fractures and 21 SC fractures were not visible on CT, but were visible on MRI (Fig. 5). There was no significant difference in fracture detection among TLJ fractures, but there was a difference in the frequency of SC fracture detection, with the difference being statistically significant in the T12 and L1 regions (*p* = 0.002, 0.014, respectively). The proportion of SC fractures at the TLJ region was also significantly increased on MRI scans compared to CT scans at the T12 (MRI 13.73%, CT 7.19%, *p* = 0.001) and L1 levels (MRI 11.76%, CT 7.84%, *p* = 0.002).

**Table 4:** Comparison of performance of CT and MRI in detecting SC fractures.

Spine level	No. of fracture	Accompanying SC fracture			*P *value
		Presence	No. of fracture (%) on MRI	No. of fracture (%) on CT	
T10	7	(−)	5 (71.43)	5 (71.43)	–
		(+)	2 (28.57)	2 (28.57)	
T11	15	(−)	10 (66.67)	13 (86.67)	0.083
		(+)	5 (33.33)	2 (13.33)	
T12	52	(−)	31 (59.62)	41 (78.85)	0.002*
		(+)	21 (40.38)	11 (21.15)	
L1	59	(−)	41 (69.49)	47 (79.66)	0.014*
		(+)	18 (30.51)	12 (20.34)	
L2	37	(−)	31 (83.78)	33 (89.19)	0.157
		(+)	6 (16.22)	4 (10.81)	

*No* number, *SC* sacrococcygeal.

*p < 0.05.

## Discussion

Two key results from this study, which aimed to identify concurrent TLJ and SC fractures using a modified MRI protocol, can be highlighted. First, using the modified MRI protocol in patients with trauma, the prevalence of concurrent TLJ and SC fractures was found to be approximately 30%. This protocol changed only the FOV without affecting the scan time. Using the existing MRI protocol, the rate of SC fractures in TL and TLJ fractures was found to be 7.58% (which was comparable to the rate of 7.1% found in a previous study^26^) and 9.02%, respectively, regardless of trauma.

There are several reasons that can explain these increased frequencies. First, the scans covered regions below S2 and a comparison of studies 1 and 2 revealed that in addition to lower SC fractures, the identification of overall SC fractures was increased. Second, we applied the common imaging modality of MRI, known to be a powerful tool for evaluating spine fractures^9,14,21,27,28^. In study 1, MRI was found to be significantly more sensitive than CT in detecting SC fractures. Several previous studies on sacral fractures have demonstrated that MRI is the most sensitive among various modalities (CT, MRI, bone scan etc.)^14,19,28^, and the CT is relatively less sensitive in detecting sacral fractures^9,10,17,25,28^. Moreover, the ACR guidelines for SIF notes that MRI is very sensitive and that it is the favored procedure rather than bone scan or CT. This is because MRI is more sensitive than CT in the evaluation of stable, non-displaced SC fractures such as SIF^25^. Most of the newly discovered SC fractures in this study were stable or lower SC fractures, and MRI would be more useful even in cases involving trauma. Third, the FS sequence increased the sensitivity of detecting occult fractures at the SC spine. The FS sequence has been known to be sensitive in detecting micro-fractures which are difficult to identify in T2WI or CT^21,29^. Fourth, in study 1, radiologists could focus on discovering SC fractures among TLJ fractures when assessing the possible presence of a tandem fracture and this may have led to the increased detection rate of concurrent fractures.

Our second key result was the finding that axial loading trauma mechanism was an important risk factor for concurrent TLJ and SC fractures. Although previous studies have not investigated the association between concurrent fractures and axial loading, there have been two related studies: one showed no statistical significance in any of the factors assessed^26^, while in the other, BMD (< 2.5) and old age were found to be risk factors^19^. However, they were not limited to patients with trauma and SC fractures were restricted to SIFs only. Many previous studies on SC fractures have mainly focused on insufficiency fractures, and the association with bone density and related factors such as age has been mentioned^9,15,19^. In our study, we could not completely separate traumatic SC fractures from SIFs which were also frequently triggered by axial loading such as falling on the buttocks^15^. Nevertheless, fractures in young patients or those with a normal bone density may be more easily explained as being traumatic. These concurrent fractures corresponded to noncontiguous multi-segment fractures and were associated with high energy injuries^30^; moreover, a high TLICS score was another factor linked to a high energy injury^7^. However, these were not found to be significant risk factors for concurrent fractures, consistent with findings from a previous study^19^.

In our study, axial loading injuries were limited to falling on the buttocks. SIF mainly occurs at the S2 region and frequently involves the sacral ala^28^; however, in our study, SC fractures were seen in the lower SC spine, consistent with previous studies^10,17^. This may be because the lower SC spine is the area that is generally directly impacted by injury. Moreover, the part under the center of gravity of the axial loading trauma moves posteriorly while the upper thorax moves forward^31,32^.

Despite a non-negligible incidence of SC fractures^9,14,15^, most SC fractures are stable and managed conservatively^9–11^. Surgery is considered for cases involving major unstable fractures, presence of neurological deficits, or severe malalignment^10^. In this study, surgery was required in just one case in study 2. In study 1, when patients with TLJ and SC fractures were followed-up, pain in the sacral area in some patients necessitated an increase in the dosage and/or duration of pain medication. Furthermore, some patients were hospitalized again because of persistent neurological symptoms such as radiculopathy in the leg. When a concurrent SC fracture is identified, delayed ambulation is recommended if the patient complains of pelvic pain with weight bearing. However, the motility restriction period of patients with concurrent TLJ and SC fractures was not different compared to only a TL fracture. Additionally, a few patients complained of pelvic dysthesia without critical bowel or urinary dysfunction. However, none of them showed any indications that warranted surgery. This might be because additional fractures in study 1 were diagnosed more often in the S4-coccyx area than in the S1–3 region. Clinically, S4-coccyx fractures are stable without the accompaniment of neurologic deficits. The diagnosis of SC fracture is correlated to clinically concealed lower back or pelvic pain in TL fractures^33^, which tends to get neglected. Furthermore, even though clinicians may suspect fractures, they are often missed on radiography and CT. However, if clinicians identify concurrent fractures using the modified MRI protocol, they can explain the cause of pain, suggest life style modifications, and consider conservative treatment^34,35^. Although most of the additional SC fractures that were detected in this study were stable, we believe that this modified MRI protocol could be very helpful in improving the quality of life of patients by helping clinicians better understand the patients’ symptoms rather than focusing solely for making decisions for surgery.

Our study has several limitations. First, this study was based on a small sample size which is not reflective of the wider community of patients. Larger, multi-centric studies may address this limitation. Second, the modified MRI protocol may incur some criticism in evaluating degenerative spine pathology. When the FOV is large, the spatial resolution decreases along with an increased signal-to-noise ratio. However, simply extending the FOV by 2–3 cm in the sagittal scans did not affect the critical evaluation of posterior ligamentous complex injuries or neurologic structures and did not have a significant effect on our diagnoses of other pathologies. Third, in this study, we did not evaluate routine thoracolumbar spine MRI scans in cases involving pelvic bone fracture if the pelvic bone fracture patient did not complain of back pain; this is because the trauma mechanism of pelvic bone fracture is widely varied from axial loading to shearing force and direct injury^18^. However, in cases involving axial loading, the possibility of concealed spine fracture(s) accompanying pelvic bone fracture cannot be discounted.

In conclusion, the frequency of concurrent SC and TLJ fractures as detected by the modified MRI protocol was approximately 30%. An axial loading mechanism of injury was the only significant risk factor associated with these concurrent fractures, regardless of osteoporosis or age. In addition, radiologists need to be mindful of the SC area when assessing TLJ fractures to improve the detection of co-existing SC fractures.
